# Unmasking neurodevelopmental assessment: the potential role for virtual reality as a multimodal clinical adjunct

**DOI:** 10.3389/frhs.2026.1927026

**Published:** 2026-10-23

**Authors:** Crystal C. W. Goh, Albert S. Rizzo

**Affiliations:** 1 Cognitive Leap Solutions Inc., Irvine, CA, United States; 2 Institute for Creative Technologies, University of Southern California, Los Angeles, CA, United States

**Keywords:** ADHD, autism, camouflaging, masking, virtual reality, neurodevelopmental assessment, psychomotor behavior, ecological validity

## Abstract

Children with neurodevelopmental conditions (NDCs), particularly attention-deficit/hyperactivity disorder (ADHD) and autism spectrum disorder (ASD), may alter or suppress aspects of their behavioral presentation in structured social and assessment contexts. This phenomenon, often described as masking or camouflaging, can complicate clinical interpretation and may contribute to delayed or incomplete identification in some individuals. Immersive virtual reality (VR) offers a potentially useful complementary assessment context by combining ecologically relevant task demands with continuous measurement of cognitive performance and objective metrics such as gaze and motor behavior that are less accessible to deliberate control. We propose that immersion in VR may also reduce the salience of the immediate social audience and thereby lessen some forms of assessment-context distortion, although this mechanism has not yet been directly tested. Emerging studies demonstrate that VR-derived motor and gaze measures can distinguish group-level neurodevelopmental profiles and provide information beyond conventional task performance. While these measures are not currently diagnostically specific, they may serve as adjunctive sources of behavioral information to support clinical decisions. Drawing on independent research and clinical implementation experience, this Perspective examines the empirical basis for VR-enabled assessment, proposes testable mechanisms by which it might reduce contextual distortion for children with NDCs, and considers the validation, ethical, accessibility, cultural, and implementation requirements necessary for responsible clinical use.

## 1 A structural gap in neurodevelopmental assessment

Neurodevelopmental conditions (NDCs), including attention-deficit/hyperactivity disorder (ADHD) and autism spectrum disorder (ASD), affect a substantial and growing proportion of children globally, with a pooled prevalence estimate of 7.2% for ADHD in children (95% CI 6.7–7.8) (1), and co-occurrence between ADHD and autism documented in up to 43.8% of autistic children (2). The clinical picture is further complicated by high rates of missed and delayed identification, particularly in girls, children from minority ethnic backgrounds, and those in under-resourced settings (3, 4). Across healthcare systems, diagnostic waiting times routinely extend to years (5, 6), and the tools most commonly used — parent and teacher rating scales and standardized performance tests — carry well-documented susceptibility to observer bias, rater expectation effects, and cultural variation in what constitutes atypical behavior (7, 8).

Immersive virtual reality (VR) has attracted growing interest as an assessment platform for NDCs, primarily on grounds of ecological validity — the argument that assessing children in environments resembling those where their difficulties most meaningfully manifest produces more representative behavioral data than stripped laboratory conditions (9, 10). Clinician-administered assessments conducted in one-on-one structured settings are particularly vulnerable to masking or camouflage — the active or habitual suppression of neurodivergent traits in response to social performance demand (11, 12) — a phenomenon well-established in the neurodiversity literature but underappreciated in clinical assessment practice. VR may offer a meaningful response to this problem, operating through two complementary mechanisms: an environmental mechanism, by partially removing the visible social audience, VR may reduce the conditions that drive masking; and a neurobiological mechanism, where VR-based assessment captures continuous motion and gaze signals that reflect atypically organized oculomotor and motor circuitry in ADHD and autism (13), and that are less accessible to deliberate control than overt task responses (14).

This Perspective examines the evidence base of VR's value in reducing context-related distortions and masking during NDC assessment, and explores the implementation barriers that determine whether VR-based NDC assessment reaches clinical scale, as well as the prospective studies required to test whether these mechanisms translate into more accurate and equitable identification of neurodivergent children. The empirical VR literature on neurodevelopmental assessment is concentrated primarily in ADHD and autism — the two conditions for which the largest bodies of behavioral, oculomotor, and motor evidence exist, and for which the neurobiological rationale developed in this Perspective is most directly supported. Findings from these populations should not be assumed to generalize to other neurodevelopmental conditions without further independent empirical validation.

## 2 Masking: when the assessment environment may be a confound

Masking, or camouflaging, refers to the active or habitual modulation of neurodivergent traits in social contexts where those traits may attract negative attention or consequence (11). It is most extensively documented in autism, where it encompasses mirroring social behavior, suppressing stimming, and scripting interactions — strategies that carry significant personal cost, including heightened anxiety, burnout, and systematically delayed identification, particularly in girls (15, 16). Camouflaging in autism has also been associated with elevated hair cortisol concentration, although this association was not confirmed within twin pairs, suggesting familial confounding (17). In autistic women, camouflaging has been linked to neural self-representation responses in ventromedial prefrontal cortex (18), and the first neurocognitive study of masking found faster N170 latencies and attenuated late positive potentials to facial emotion in adolescents who passed as non-autistic (19). Evidence that camouflaging extends to ADHD is emerging, with adults with ADHD reporting more camouflaging than comparison participants but less than autistic adults, and with autistic traits rather than ADHD traits predicting camouflaging (20), and qualitative evidence suggesting that adolescent girls with ADHD engage in sustained impression management and behavioral suppression within school contexts (21). The pediatric evidence base remains considerably more limited for ADHD than for autism, and the neurobiological mechanisms underlying camouflaging in ADHD are less well characterized. Across both conditions, however, the functional outcome suggests that a child may present as more regulated during assessment than their daily life may indicate.

The clinical assessment setting embodies precisely the conditions that drive masking — structured novelty, an attentive adult examiner, and implicit performance demand — making it the environment most likely to elicit effortful self-regulation in children who have learned to suppress their natural presentation. For girls and women with ADHD and autism in particular, years of social learning and deliberate effort to appear neurotypical may produce a surface presentation that substantially misleads observers not specifically trained to identify masked profiles (3, 22). Parental presence compounds this dynamic, as children may perform differently on neuropsychological tests when a parent is present as an observer (23).

The consequence is a potential structural decoupling between what the assessment captures and the child's most functionally relevant behavioral repertoire. A child who struggles daily with attentional regulation, sensory overwhelm, and impulse control may present as organized and engaged across a forty-minute clinical evaluation, whereby the assessment context may elicit the child's most regulated performance. Whether reducing the salience of that social-performance context would reveal behavioral information that complements conventional assessment is a hypothesis that requires further research. No study to date has directly demonstrated that children camouflage less in immersive VR than during conventional clinician-administered assessment, and establishing this will require direct within-person comparisons across contexts, with simultaneous measurement of masking behavior, observer effects, and alternative explanations including novelty and prior VR experience.

## 3 Unmasking with VR? Two complementary mechanisms

VR's clinical value in neurodevelopmental assessment may reach beyond ecological validity, with two specific mechanisms proposed here that address the unique qualities of NDCs. The first is the partial removal of the social audience during assessment. Once a child is immersed in a virtual environment via a head-mounted display (HMD), the physical presence of the examiner recedes from the child's perceptual field, potentially reducing one source of social-performance demand. Research using immersive VR with children suggests that virtual environments may elicit more naturalistic behavior than conventional testing conditions, particularly for tasks involving inhibitory control and attention (24, 25). A scoping review of immersive VR assessment across NDCs found the field concentrated in autism and ADHD, with ADHD studies focused primarily on the assessment of executive performance (26). While this does not assume that masking disappears entirely in VR — motivated, experienced maskers may maintain some degree of suppression — the salience of one hypothesized contributor to assessment-context distortion may be reduced.

The second mechanism is the capture of motor behavior as a distinct assessment channel. VR-delivered assessment via HMDs can capture continuous head, body, and gaze movement data alongside cognitive task performance, simultaneously within the same session, without additional hardware. Continuous motor behavior provides an additional measurement channel that may be less readily controlled than explicit task responses. ADHD is associated with alterations across the cortico-striato-thalamo-cerebellar (CSTC) circuit, the subcortical architecture coordinating cognitive intention with motor action (27–30). Atypical striatal dopamine signaling has been linked to characteristic psychomotor patterns — elevated movement frequency, expanded spatial range, reduced stillness, and diminished orientation toward task-relevant stimuli — although dopamine transporter findings are mixed and partly influenced by prior stimulant exposure (31, 32). From an assessment perspective, the value of VR-based motor measurement lies less in treating movement as a specific biomarker than in capturing continuous behavioral variation that conventional cognitive testing often leaves unmeasured.

Motor differences are not unique to ADHD among NDCs. Motor difficulties are increasingly recognized as a common feature of autism, with differences in coordination, gait, and postural stability documented across meta-analytic and large-cohort studies (33, 34). These signatures can be captured in VR environments using the same tracking infrastructure applied to cognitive assessment, making motor behavior a shared, cross-diagnostic signal that standardized tools currently do not measure.

## 4 A converging evidence base

Converging evidence supports the feasibility of capturing these signals within immersive VR. Eye movement parameters — saccadic latency, fixation stability, and gaze orientation — are sensitive, objective, and less accessible to deliberate control than overt task responses (14). Merzon et al. (35) demonstrated that gaze behavior during a VR prospective memory task distinguished children with ADHD from neurotypical controls, with a classifier trained on eye-movement features reaching an area-under-curve (AUC) value of 0.92. Stokes et al. (36) showed that distractor presence disrupted on-task gaze in children with ADHD even when the distractor itself was not fixated — indicating that attentional disruption in naturalistic contexts is captured in gaze dynamics in ways that task accuracy alone cannot reveal. Eye tracking combined with conventional continuous performance test (CPT) measures outperforms either modality alone, with integrated approaches achieving AUC values of 0.889 compared to 0.769 for CPT alone (37), and modern standalone HMDs increasingly incorporate integrated eye trackers without additional hardware.

Full-body motion tracking extends the motor signal beyond head movement. Jeong et al. (38) found that, in children with ADHD assessed in an immersive VR environment, total distance traveled was the strongest single predictor of hyperactivity severity, while average movement speed showed the broadest associations across symptom dimensions. Hocking et al. (39) demonstrated that low-cost motion capture integrated with a VR environment could quantify whole-body movement efficiency, synchrony, and symmetry in autistic youth, although in a small feasibility sample. VR- and machine-learning-based approaches are characterized as promising but still emerging, with generally small and heterogeneous studies and unresolved questions regarding clinical validity, generalizability, and interpretability (40).

Measurable motor or gaze differences should not be interpreted as diagnosis-specific biomarkers. Similar behavioral features may arise from attentional regulation, motor coordination, anxiety, sensory characteristics, medication effects, or familiarity with the technology. The present value of multimodal VR measurement is better framed as richer behavioral characterization than as identification of disorder-specific signatures, and diagnostic specificity remains an empirical question requiring direct comparison across multiple neurodevelopmental groups.

The Virtual Classroom Assessment Tracker (vCAT™) represents one implementation of this paradigm, grounded in a normative dataset of 837 neurotypical children aged 6–13 across seven US clinical sites (41). Administered via an HMD in a simulated school classroom, the vCAT simultaneously captures twelve cognitive and motor metrics across a 13-minute AX-continuous performance task (AX-CPT) session, with cognitive and motor domains representing two signal types that reflect distinct behavioral domains. Sex differences in the neurotypical sample — boys demonstrating greater motor activity and more impulsive responding, girls demonstrating earlier inhibitory regulation — carry direct implications for diagnostic equity, offering a normative framework that may help characterize behavioral profiles that are less readily captured through observer-based assessment alone. Preliminary clinical comparison data from children with confirmed ADHD profiles show broad-spectrum group differences characterized by elevated intra-individual variability alongside directional performance differences, consistent with models linking intra-individual variability to dopaminergic signaling in ADHD (42, 43).

Of particular relevance to the unmasking argument are the sex differences observed across the two samples. In the neurotypical normative sample, boys demonstrated significantly greater motor activity and more impulsive responding than girls — a pattern consistent with well-documented sex differences in NDC presentation. In the ADHD sample, however, this sex differentiation appeared to converge across most motor metrics in preliminary data, with boys and girls showing broadly equivalent psychomotor profiles (43). This convergence raises the possibility that continuous motor measurement may provide information less influenced by the sex-typed behavioral expectations that affect observer-based assessment. Whether such measures improve identification of highly compensating or masking individuals, however, remains to be tested directly.

## 5 From validation to implementation, from lab to society

The clinical VR literature is rich in proof-of-concept studies and growing in validation data. It is considerably less forthcoming on what implementation actually requires, and the gap between validation and clinical scale is where most promising tools fail to reach the children they are designed to serve (Table 1).

**Table 1:** Key implementation and ethical challenges for clinical VR-based neurodevelopmental assessment.

Barrier/Risk	Why it matters	Proposed mitigation	Primary responsibility
Privacy/cybersecurity	Gaze and movement create high-dimensional behavioral records	Data minimization, encryption, access controls, retention limits	Developer + healthcare organization
Data ownership/secondary use	Pediatric data may be reused beyond original assessment purpose	Explicit consent/assent, transparent ownership and reuse policies	Organization + developer + IRB/governance
Algorithmic opacity/bias	Scores may perform differently across demographic or clinical groups	Explainable outputs, subgroup validation, clinician review	Developer + researchers + clinicians
Hardware/software updates	Sensor or firmware changes may alter measurement properties	Version control and revalidation	Developer + regulator/health system
Cost/licensing	Hardware, software, support, and replacement costs may impede scale	Health-economic evaluation and sustainable procurement models	Payers + organizations + vendors
Sensory/physical accessibility	Some children may not tolerate an HMD or standard interaction	Familiarization, alternative pathways, clear stopping criteria	Clinicians + developers
Cultural/language validity	Classroom design, instructions, and norms may not generalize	Cultural co-design, translation, local validation and representative norms	Researchers + communities + developers
Workforce integration	Clinicians need skills in administration and interpretation	Training, competency standards, workflow integration	Professional bodies + organizations

HMD, head-mounted display; IRB, institutional review board; VR, virtual reality.

### 5.1 Intended clinical role and developmental calibration

At the current stage of evidence, VR-based neurodevelopmental assessment may best be positioned as an adjunctive source of standardized behavioral information. VR-derived measures of attention, motor activity, and gaze can complement developmental history, clinical interview, parent and teacher report, and validated rating scales, while the clinician remains responsible for integrating these sources for clinical decision-making.

A constraint specific to pediatric NDC assessment is the pace of neurological development across the school-age range. A child's psychomotor and attentional profile shifts substantially between ages 6 and 13, with the largest group differences observed in younger children and a pattern of convergence in adolescence consistent with delayed but ultimately achieved maturation (43). Normative adequacy is therefore not a one-time validation requirement but an ongoing developmental calibration challenge, and tools validated on one age band should be applied cautiously outside it. The children most likely to be misidentified are also those whose developmental trajectories are least well represented in existing normative databases.

### 5.2 Clinician training, accessibility, and population fit

The clinician's role in VR-delivered assessment shifts from test-administrator to environment-manager and data-interpreter — competencies that pre-licensure training does not yet address. Training practitioners to understand not only the administration protocol but the theoretical rationale for what is being measured — why motor and gaze signals are clinically informative, how they relate to the neurobiological profiles underlying NDCs, and how to communicate findings to families — is as demanding as any technical aspect of implementation. A practitioner who understands why the motor channel may be less susceptible to masking than behavioral presentation is better positioned to interpret and communicate VR-derived data than one who understands only how to administer the task.

Population fit is rarely as clean as validation samples suggest, and this problem is more pronounced in neurodivergent pediatric populations specifically. Autistic children may disproportionately find HMD wearing intolerable or become dysregulated by aspects of the virtual environment — the same sensory sensitivities that contribute to masking in conventional assessment settings may also affect engagement with VR. Designing for sensory accessibility and structured pre-assessment familiarization protocols is therefore a prerequisite for reaching the population for whom the unmasking argument is most relevant.

### 5.3 Cultural adaptation, equity, and sustainability

Family attitudes toward HMD technology vary across cultural contexts, and the cultural frameworks through which families understand neurodevelopmental difference shape how VR-derived findings are received and acted upon. The populations most underserved by existing NDC assessment infrastructure — children from minority ethnic backgrounds, those in under-resourced settings, those whose profiles are least legible to observer-based tools, and the children least likely to reach the specialists who use them — are precisely those for whom normative breadth, cultural adaptation, and equitable access to VR-based assessment matter most. Building this infrastructure requires investment in the surrounding ecosystem — training pipelines, culturally adapted normative databases, and reimbursement pathways — directed with the same rigor applied to the technology itself.

## 6 Limitations and research agenda

The central unmasking proposition advanced in this Perspective remains a hypothesis. Existing studies demonstrate that immersive VR can generate ecologically situated cognitive, motor, and gaze measures and can distinguish groups on selected behavioral dimensions. Whether children engage in less masking in VR than during conventional assessment has not yet been directly demonstrated, and behavioral differences observed in immersive environments may alternatively reflect novelty, gaming experience, altered motivation, sensory characteristics, or greater task engagement. Most available evidence concerns ADHD and autism, and the diagnostic specificity of motor and gaze measures across neurodevelopmental and psychiatric conditions remains uncertain.

Direct testing of the proposed mechanism will require within-participant studies comparing conventional and immersive assessment conditions, ideally with randomized order and experimental manipulation of observer presence. Such studies should measure camouflaging directly, assess gaming and VR familiarity, quantify cognitive, gaze, and motor behavior simultaneously, and examine whether VR-derived information improves blinded clinical classification beyond established assessment methods. Multidiagnostic studies are needed to determine whether candidate behavioral markers provide disorder-specific information or index transdiagnostic dimensions such as attentional regulation, motor coordination, or arousal.

Prospective multisite studies should establish test–retest reliability, developmental norms, demographic and cultural generalizability, accessibility, patient and family acceptability, clinician usability, incremental diagnostic value, and cost-effectiveness. Independent replication will be particularly important where systems being evaluated are developed or commercially supported by investigators involved in the research.

## 7 Discussion

Many neurodivergent children have learned, at significant personal cost, to suppress the presentations that most honestly reflect their neurological profiles, and standard assessment environments tend to elicit that suppression rather than reduce it (11, 12). VR assessment, by partially removing the social audience and by capturing motor and gaze signals that are less accessible to deliberate control than overt task responses, may offer a measurement context better aligned with the behaviors of clinical interest. Whether this translates into reduced masking, improved diagnostic accuracy, or more equitable identification remains a hypothesis requiring direct empirical test.

Converging findings across head movement, gaze, and full-body motion capture support VR as a viable platform for capturing behavioral profiles that standard methods may miss. The literature presently supports feasibility and measurement potential — not yet incremental diagnostic value, and not yet demonstrated reduction in masking. The prospective studies required to establish these effects have not been conducted.

Whether these tools reach clinical scale will be determined by evidence of effectiveness, clinician training, cultural adaptation, sensory accessibility, normative breadth, and reimbursement policy. That question — whether immersive measurement can reach what masking conceals — is both testable and worth testing.

## Data Availability

Publicly available datasets were analyzed in this study. This data can be found here: this article is a Perspective and does not report primary data collection. Normative data supporting findings discussed in this article are available in Goh et al. (2024) (41), Frontiers in Virtual Reality. Clinical comparison data referenced as Goh & Rizzo (2026) (43) are contained in a manuscript currently under review and will be made available upon publication.
